# Galectin-3 Mediates Thrombin-Induced Vascular Smooth Muscle Cell Migration

**DOI:** 10.3389/fcvm.2021.686200

**Published:** 2021-10-20

**Authors:** Lei Tian, Chun-Kai Huang, Fenghua Ding, Ruiyan Zhang

**Affiliations:** Department of Cardiovascular Medicine, Ruijin Hospital, Shanghai Jiao Tong University School of Medicine, Shanghai, China

**Keywords:** thrombin, galectin-3, VSMCs, migration, signaling pathway

## Abstract

Vascular smooth muscle cell (VSMC) migration is an important step in the progression and development of vulnerable plaques. Thrombin is involved in both physiological and pathological processes of atherosclerosis. Therefore, the elucidation of the mechanisms underlying thrombin-induced VSMC migration is essential for devising effective treatments aimed at the prevention of plaque instability. In this study, we found that thrombin activated MAPK signaling pathways and increased the expression of galectin-3, which was also a well-known factor in atherosclerosis. Knockdown of galectin-3 by specific small interfering RNA (siRNA) blocked thrombin-induced activation of ERK1/2 and p38 MAPK, but not JNK MAPK. Src/FAK phosphorylation was also shown to be activated by thrombin. FAK autophosphorylation at Y397 was most significantly inhibited by galectin-3 siRNA. Galectin-3 siRNA or specific inhibitor (P38 MAPK inhibitor and ERK1/2 inhibitor) effectively prevented thrombin-induced VSMC migration via reducing paxillin expression. These findings demonstrate, for the first time, that thrombin stimulation of VSMC migration and paxillin expression are regulated by galectin-3, and ERK1/2, p38 MAPK, and Src/FAK signaling pathways are involved in this process. These results are beneficial to clarify the role of galectin-3 in thrombin-induced advanced lesions in atherosclerosis and shed new insights into the regulatory mechanism of VSMC migration in combating plaque rupture.

## Introduction

The incidence of atherosclerosis is increasing over the developing countries. Acute coronary syndrome (ACS) including unstable angina, non-ST elevated myocardial infarction (MI), or ST-elevated MI is associated with substantial morbidity and mortality in coronary heart diseases (1). The unstable atherosclerotic lesions followed by plaque rupture are the primary causes of ACS (2, 3). Vascular smooth muscle cells (VSMCs) are the principal cellular components in the medial layer of arteries. The transformation of VSMCs from a contractile phenotype to a synthetic phenotype is considered as a typical characteristic in atherosclerosis. VSMC proliferation and migration is a key event in the pathogenesis of atherosclerosis. In the plaque, the majority of VSMCs are derived from the medial layer of artery. In the plaque, the majority of VSMCs are derived from the medial layer of artery, these VSMCs with high ability of proliferaion and migration can migrate to the intima. During this process, VSMCs migrate from the medial layer to intima to accelerate the progression and development of vulnerable plaques (4).

Thrombin, a serine proteinase, activates protease-activated receptors (PARs) by its extracellular N-terminal domain and unmasking a tethered ligand (5, 6). As a critical enzyme in coagulation systems, thrombin can convert fibrinogen into fibrin, which constitutes the thrombus core (7). Thrombin enhances migration of VSMCs, which has a central place in plaque instability and vascular remodeling. Several signaling pathways have been found to mediate thrombin-induced cell migration. Thrombin-stimulated mitogen-activated protein kinase (MAPK) activation has been shown to initiate cell migration (8). Thrombin increases VSMC migration via P38-MAPK signaling pathways (9). Thrombin-mediated PAK1 activation plays a key role in VSMC migration (10). Despite advances in the role of thrombin in cell migration, the precise mechanisms of VSMC migration induced by thrombin remain largely unclear. Therefore, exploring the possible molecular mechanism underlying thrombin-induced vascular remodeling has important significance for preventing and treating atherosclerosis.

Galectin-3 (Gal-3), a 29- to 35-kDa protein, is a member of the β-galactoside-binding lectin family (11). Gal-3 is widely spread in heart, vessels, lung, bone, muscle, liver and kidney, and related to fibrosis (11, 12). In the coronary heart disease patients, gal-3 was proved to be an important prognostic biomarker in MI (13). Gal-3 level increases in patients with ischemic heart disease; besides, there is a significant relationship between gal-3 level, MI size, and left ventricle (LV) remodeling (13, 14). Acute MI increases gal-3 level, which is positively correlated with left ventricular ejection fraction (LVEF) (14, 15). Besides, in our previous research, gal-3 was found to promote VSMC migration via the Wnt/β-catenin signaling pathway (16).

Until now, the role of gal-3 in physiological and pathological processes of atherosclerosis is still unclear; in particular, we still do not know how gal-3 regulates thrombin-induced VSMC activation. The aim of this study was to explore the role of gal-3 in thrombin-induced VSMC migration. In this study, we examined gal-3 expression in thrombin-stimulated VSMCs. The activation of related signaling pathways was also investigated to determine the probable regulatory mechanisms.

## Methods

### Reagents

Dulbecco's modified eagle's medium (DMEM), fetal bovine serum (FBS), and penicillin/streptomycin were purchased from Gibco (Carlsbad, CA, USA). Thrombin, PD98059 (MAPKK inhibitor) and SB203580 (p38 MAPK inhibitor) were purchased from Sigma (Burlington, MA, USA). The primary antibodies against phospho-ERK (cat. no.4370), ERK (cat. no.4695), phospho-JNK (cat. no.4668), JNK (cat. no.9252), phospho-P38 (cat. no.8690), P38 (cat. no.9218), phospho-FAK (Tyr 397) (cat. no.3283), phospho-FAK (Tyr 576/577) (cat. no.3281), phospho-FAK (Tyr 925) (cat. no.3284), FAK (cat. no.3285), p-Src (Tyr 416) (cat. no.6943), Src (cat. no.2109), and GAPDH (cat. no.5174) were acquired from Cell Signaling Technology, Inc. (Danvers, MA, USA). Anti-gal-3 (cat. no. ab76245) and anti-p-paxillin (cat. no. ab4832) were obtained from Abcam (Cambridge, UK). A Cell Counting kit-8 (CCK-8) assay was purchased from Dojindo Molecular Technologies, Inc. (Kumamoto, Japan). All other chemicals were from commercial sources.

### Cell Culture

Primary coronary artery smooth muscle cells were obtained from the American Type Culture Collection (ATCC, Rockville, MD, USA). VSMCs were maintained in DMEM supplemented with 10% FBS and 1% penicillin/streptomycin at 37°C, 5% CO_2_ incubator. In order to allow cells into the relatively same condition, VSMCs were cultured in DMEM without serum for 12 h before further experiments. We performed our further experimentations by using the cells at a density of 10 (5)/well in six-well-plates.

### SiRNA Interference

siRNA method was performed to inhibit gal-3 expression. Briefly, 5 × 10 (5) VSMCs per well were cultured in six-well-plates to 75% confluence. The cells were then transfected with gal-3 siRNA with Lipofectamine^®^ 2000. The process of transfection was performed in the absence of antibiotics. Following 48 h incubation, the cells were used for other experiments. The human gal-3 siRNA sequence was 5'-CCUCGCAUGCUGAUAACAATT-3', and the scrambled siRNA sequence was 5'-UUGUUAUCAGCAUGCGAGGTT-3'. siRNA was synthesized by Biotend (Shanghai, China).

### Migration Assay

Cell migration was analyzed by transwell method (17, 18). Briefly, following cellular transfection with gal-3 siRNA or scramble siRNA, VSMCs were resuspended in DMEM and loaded into the upper chambers (Corning Inc., Corning, NY, USA). The lower chambers were filled with DMEM in the presence or absence of 2 U/ml thrombin. After 24-h incubation for 37°C, the lower side of the filter was washed and fixed prior to being stained with 4′,6-diamino-2-phenylindole (DAPI; 1:1,000; Sigma-Aldrich) for 5 min. The cells were counted using a microscope in three random high-power fields (magnification, ×100) for each well.

### qRT-PCR

Total RNA was extracted using TRIzol^®^ reagent. Total RNA was reverse-transcribed into cDNA, and RT-qPCR was performed on an Applied Biosystems 7500 Real-Time PCR system (Applied Biosystems Life Technologies, Foster City, CA, USA) by using SYBR Premix Ex Taq II and gene-specific primers. The primers targeting human gal-3 and GAPDH were as follows: Gal-3, forward 5′-GGCCACTGATTGTGCCTTAT-3′, and reverse 5′-TGCAACCTTGAAGTGGTCAG-3′; GAPDH, forward 5′-TGATGACATCAAGAAGGTGGTGAAG-3′, and reverse 5′-TCCTTGGAGGCCA TGTGGGCCAT-3′.

### Western Blot

Cells were lysed with 100 mM phenylmethanesulfonyl fluoride. Protein concentrations were measured with the BCA Protein Assay. The lysates (20 μg) were electrophoresed on 10% SDS-PAGE and transferred to nitrocellulose membranes (Merck Millipore, Danvers, MA, USA). The membrane was blocked with 5% non-fat dry milk in TBST buffer (100 mM NaCl, 10 mM Tris-HCl, pH 7.4, and 0.1% Tween-20) for 1 h at room temperature. The membrane was then incubated with diluted primary antibodies (1:1,000) at 4°C overnight, and then washed twice with TBST buffer and incubated for 1 h with secondary antibody at room temperature. ImageJ was used to quantity the protein by assessing band intensity.

### Statistical Analysis

Data were presented as the mean ± SEM and analyzed by one-way ANOVA followed by the Student–Newman–Keuls *post-hoc* analyses when appropriate. Non-parametric ANOVA (Kruskal–Wallis test) was used when the data did not pass the normality test. *p* < 0.05 was considered statistically significant. All experiments were performed at least three times.

## Results

### Thrombin Induced Gal-3 Expression in VSMCs

**Figure 1:**
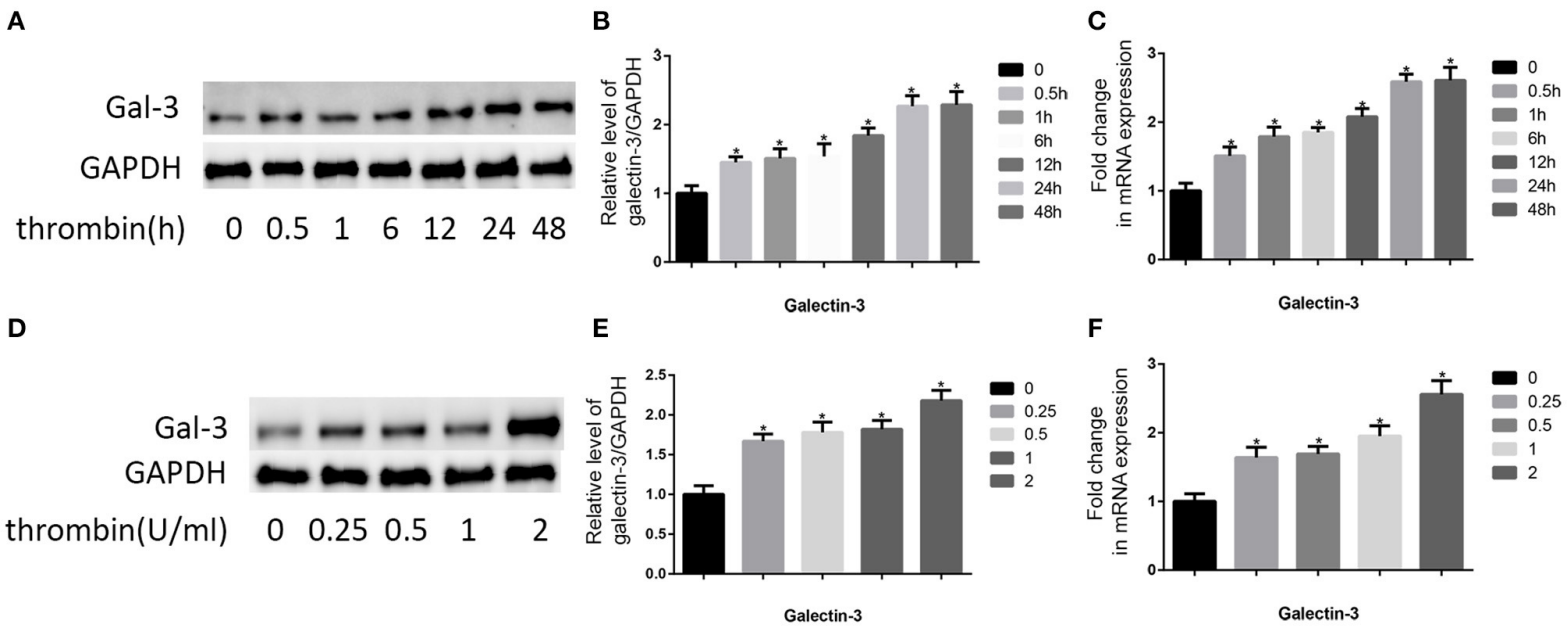
Thrombin increased gal-3 expression in VSMCs and cell migration. Cells were treated with thrombin over a range of concentrations (0, 0.25, 0.5, 1, and 2 U/ml) for different times (0, 0.5, 1, 6, 12, 24, and 48 h) and gal-3 expression was measured by Western blot **(A,D)** or qRT-PCR **(C,F)**. Quantification of Western blot results is shown **(B,E)**. Band density of native VSMCs was defined as a control and considered to 1. *p < 0.05 compared with control.

The pivotal role of gal-3 in atherosclerosis is well-known (19). Therefore, we analyzed the effect of thrombin on gal-3 expression. VSMCs were cultured in DMEM without serum for 12 h, after that, we used 2 U/ml thrombin to deal with VSMCs for different times (0, 0.5, 1, 6, 12, 24, and 48 h), besides, we also used different concentration of thrombin (0, 0.25, 0.5, 1, and 2 U/ml) to deal with VSMCs for 24 h, and then we observed gal-3 expression by using qRT-PCR and WB (Figure 1). We found that thrombin induced gal-3 expression in a time- and concentration-dependent manner.

### Gal-3 Mediated Thrombin-Induced VSMC Migration

**Figure 2:**
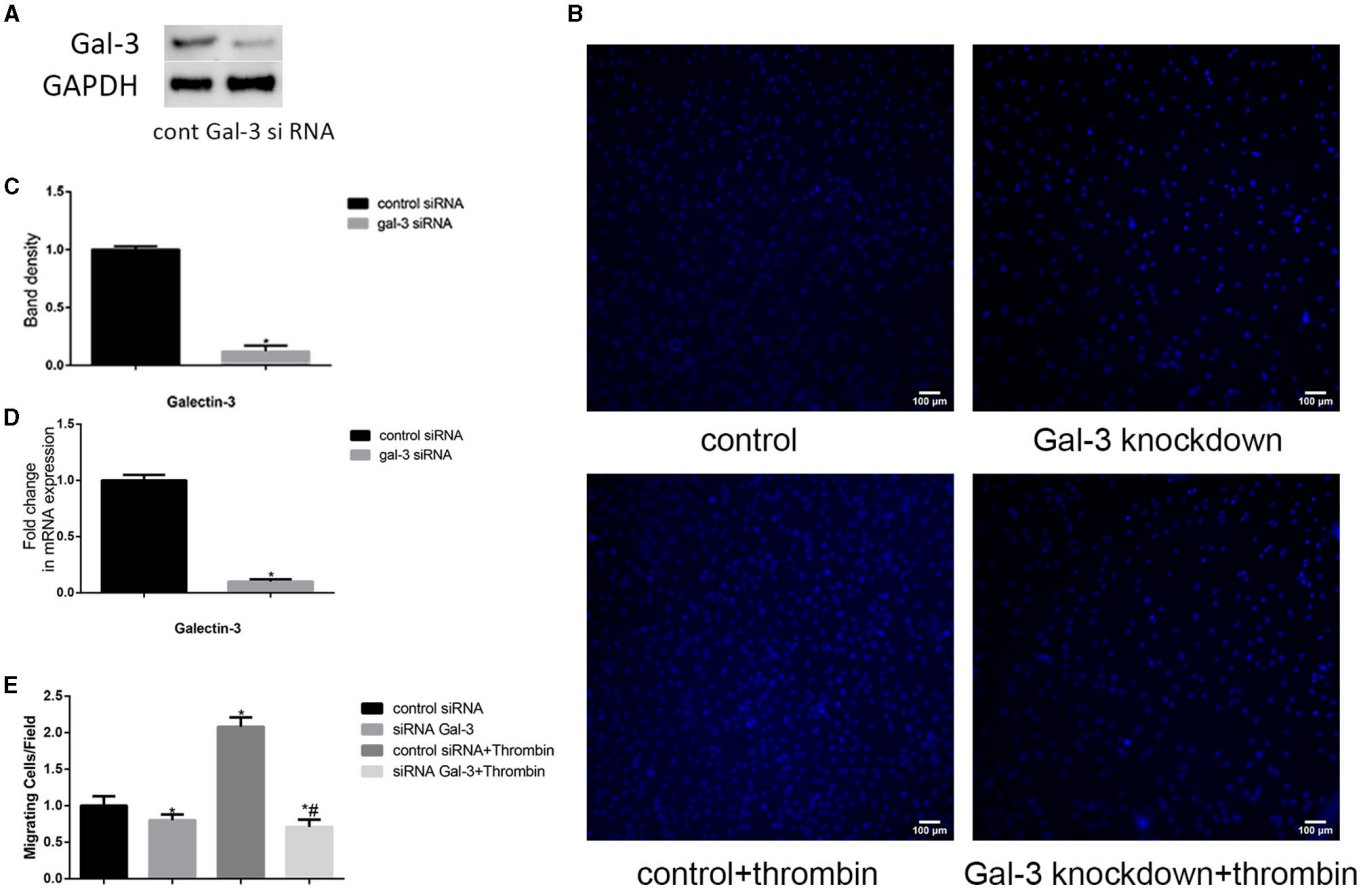
Gal-3 mediated thrombin-induced VSMC migration. VSMCs were transfected with gal-3 siRNA for 24 h. The mRNA and protein expression levels of gal-3 were measured by qRT-PCR and Western blotting separately. Western blot and qRT-PCR results of gal-3 are shown **(A,C,D)**. Band density of VSMCs transfected with scramble siRNA was chosen as a reference and set to 1. *p < 0.05 vs. the control. After transfection with either control or gal-3 siRNA for 24 h, VSMCs were added in the upper chamber. After treatment with thrombin (2 U/ml) for 24 h in the lower chamber, migrated cells from the upper chamber to the lower surface were counted in five non-overlapping fields under a microscope (×100) **(B,E)**. These blue stains are the nucleus of VSMCs that were stained with DAPI. Control siRNA transfected VSMCs were chosen as a reference and set to 1. *p < 0.05 vs. control; ^#^*p* < 0.05 vs. thrombin.

Thrombin induces a variety of proteins, which triggers coagulation systems, increases vascular permeability, and induces granulocyte chemotaxis (20). Here, we observed the effect of thrombin on VSMC migration. First, VSMCs were cultured in DMEM without serum for 12 h. After that, the serum-starved VSMCs were treated with thrombin (2 U/ml) for 24 h. As shown in Figure 2B, thrombin treatment significantly promotes VSMC migration.

In order to investigate whether gal-3 was involved in thrombin-induced VSMC migration, we used siRNA method to knock down gal-3 expression; following transfection with siRNA, gal-3 expression was significantly inhibited (Figures 2A,C,D). Migration assays were then performed to determine the effects of gal-3 inhibition on thrombin-induced cell migration. After 2 U/ml thrombin treatment for 24 h, migrated cells from the upper chamber to the lower surface were counted. Our study demonstrated that silencing gal-3 reduced cell migration and thrombin-induced VSMC migration (Figures 2B,E). These results suggest that gal-3 mediates thrombin-induced migration of VSMCs.

### ERK/P38 Signaling Pathway Mediated Thrombin-Induced VSMC Migration

**Figure 3:**
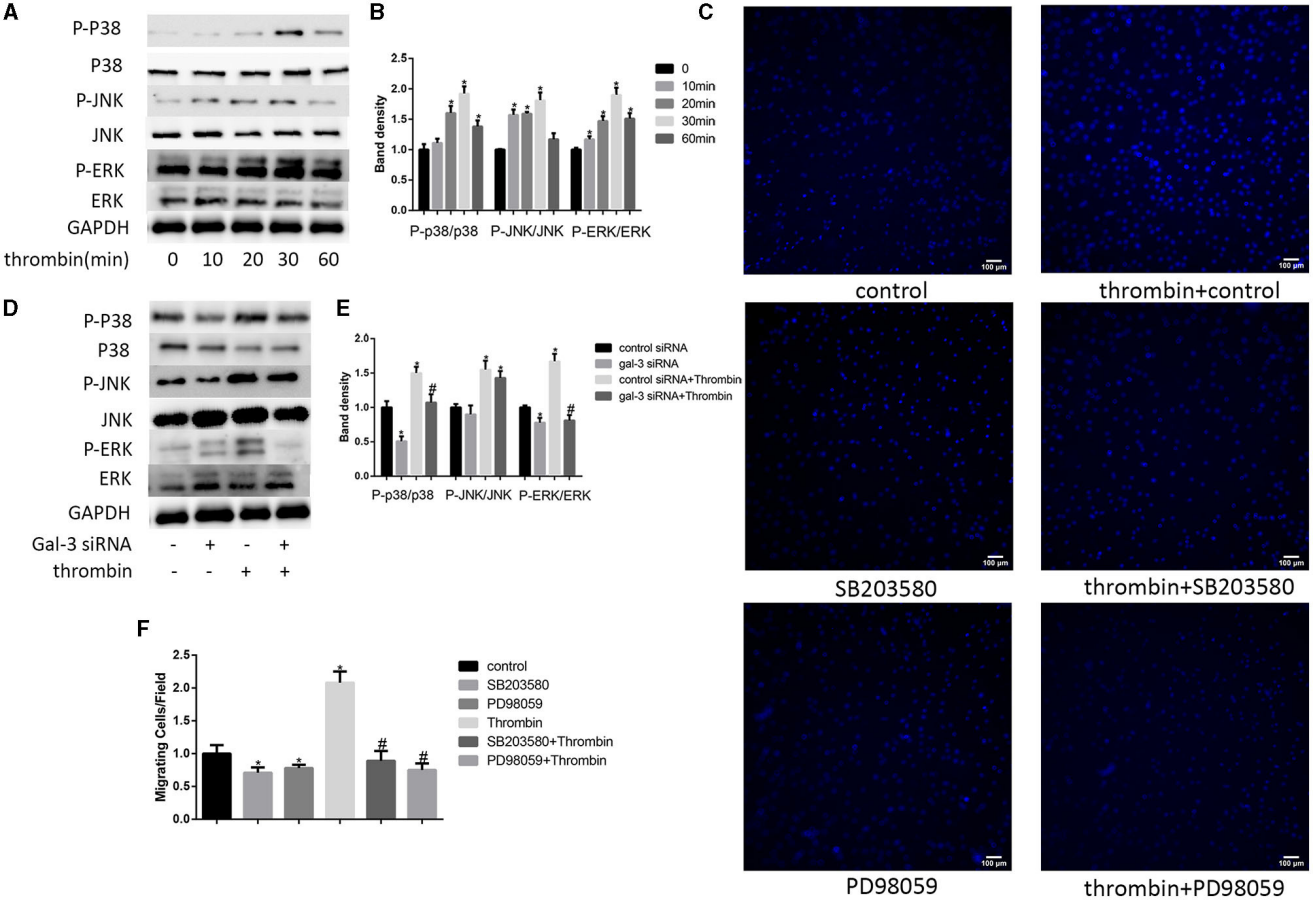
ERK/P38 signaling pathway mediates thrombin effects in VSMCs. Time dependence of thrombin-mediated MAPK signaling pathway activation in VSMCs. Cells were treated with 2 U/ml thrombin over a range of times (0–60 min), and the expression of JNK, p-JNK, ERK, p-ERK, p38, and p-p38 was measured by Western blot **(A,B)**. After transfection with either control or gal-3 siRNA for 24 h, VSMCs were incubated for 30 min in the absence or presence of thrombin (2 U/ml), and the expression of JNK, p-JNK, ERK, p-ERK, p38, and p-p38 was measured by Western blot again. Western blot results are shown **(D,E)**. Quantification of the results is given in the right panel. Band density of native VSMCs was defined as a control and considered to 1. *p < 0.05 compared with control. After incubated with either SB203580 or PD98059, VSMCs were added in the upper chamber. After treatment with thrombin (2 U/ml) for 24 h in the lower chamber, migrated cells from the upper chamber to the lower surface were counted in five non-overlapping fields under a microscope (×100) **(C,F)**. Native VSMCs were chosen as a reference and set to 1. *p < 0.05 vs. control; ^#^*p* < 0.05 vs. thrombin.

Previous studies indicate that MAPK signaling pathways regulate cell migration (21). In our research, we used 2 U/ml thrombin to treat VSMCs over a range of times (0–60 min); we found that thrombin significantly stimulated ERK, p38, and JNK in VSMCs (Figures 3A,B). To further explore whether gal-3 was involved in thrombin-induced MAPK signaling pathway activation, VSMCs were first interfered with gal-3 siRNA for 24 h before incubating with thrombin for 30 min. We found that gal-3 inhibition significantly prevented the phosphorylation of ERK1/2 and p38, but had little effect on JNK activation (Figures 3D,E). These results indicated that gal-3 mediated thrombin-induced phosphorylation of ERK1/2 and p38. These two signaling pathways are both involved in cell migration. We next further examined VSMC migration after treatment with ERK1/2-specific inhibitor (PD98059) or p38-specific inhibitor (SB203580). After incubating with either SB203580 or PD98059, VSMCs were added in the upper chamber. After treatment with thrombin (2 U/ml) for 24 h in the lower chamber, migrated cells from the upper chamber to the lower surface were counted. As shown in Figures 3C,F, ERK1/2 and p38-specific inhibitor obviously reduced VSMC migration; furthermore, increase in VSMC migration induced by thrombin was also abrogated by ERK1/2 or p38-specific inhibitor.

### Thrombin Promoted Paxillin Expression via ERK/P38 Signaling Pathway

**Figure 4:**
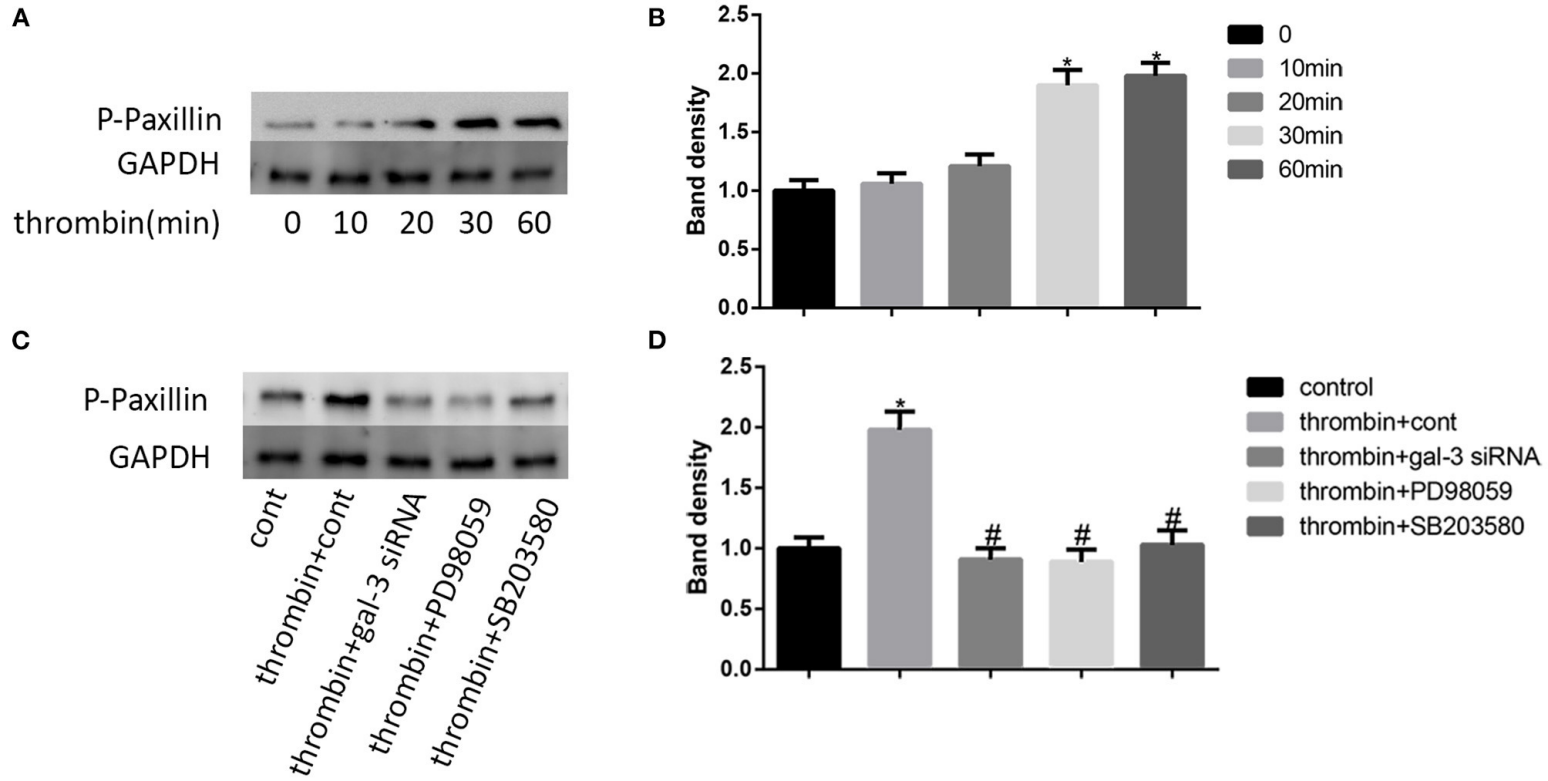
Thrombin promoted paxillin phosphorylation via ERK/P38 signaling pathway. VSMCs were treated with thrombin (2 U/ml) for different times (0, 10, 20, 30, and 60 min) and the expression of p-paxillin was measured by Western blot **(A,B)**. Cells were treated with gal-3 siRNA, PD98059, or SB203580, and then incubated in the absence or presence of thrombin (2 U/ml). The expression of p-paxillin was measured by Western blot **(C,D)**. Quantification of the results is shown in the right panel. Band density of native VSMCs was defined as a control and considered to 1. *p < 0.05 vs. control; ^#^*p* < 0.05 vs. thrombin.

Paxillin, a focal adhesion-associated protein, mediates the protein–protein interaction and regulates cell dynamic migration (22, 23). In our research, VSMCs were treated with thrombin (2 U/ml) for different times (0, 10, 20, 30, and 60 min) and the expression of p-paxillin was measured by Western blot. We found that thrombin enhanced paxillin phosphorylation at the tyrosine 31 in a time-dependent manner (Figures 4A,B). In order to furtherly explore the role of gal-3 and its downstream signaling pathways (ERK and p38 signals) in thrombin-induced paxillin phosphorylation. We specifically knocked down gal-3 expression or blocked ERK/P38 signaling pathways. VSMCs were treated with gal-3 siRNA for 24 h, or PD98059 (ERK inhibitor) or SB203580 (p38 MAPK inhibitor) for 1 h and then stimulated with 2 U/ml thrombin for 30 min. The phosphorylation of paxillin can be effectively inhibited by gal-3 siRNA, PD98059, or SB203580, suggesting that ERK/P38 inhibition results in the decrease of thrombin-induced paxillin phosphorylation (Figures 4C,D).

### Thrombin Activated Distinct FAK Signaling via Gal-3

**Figure 5:**
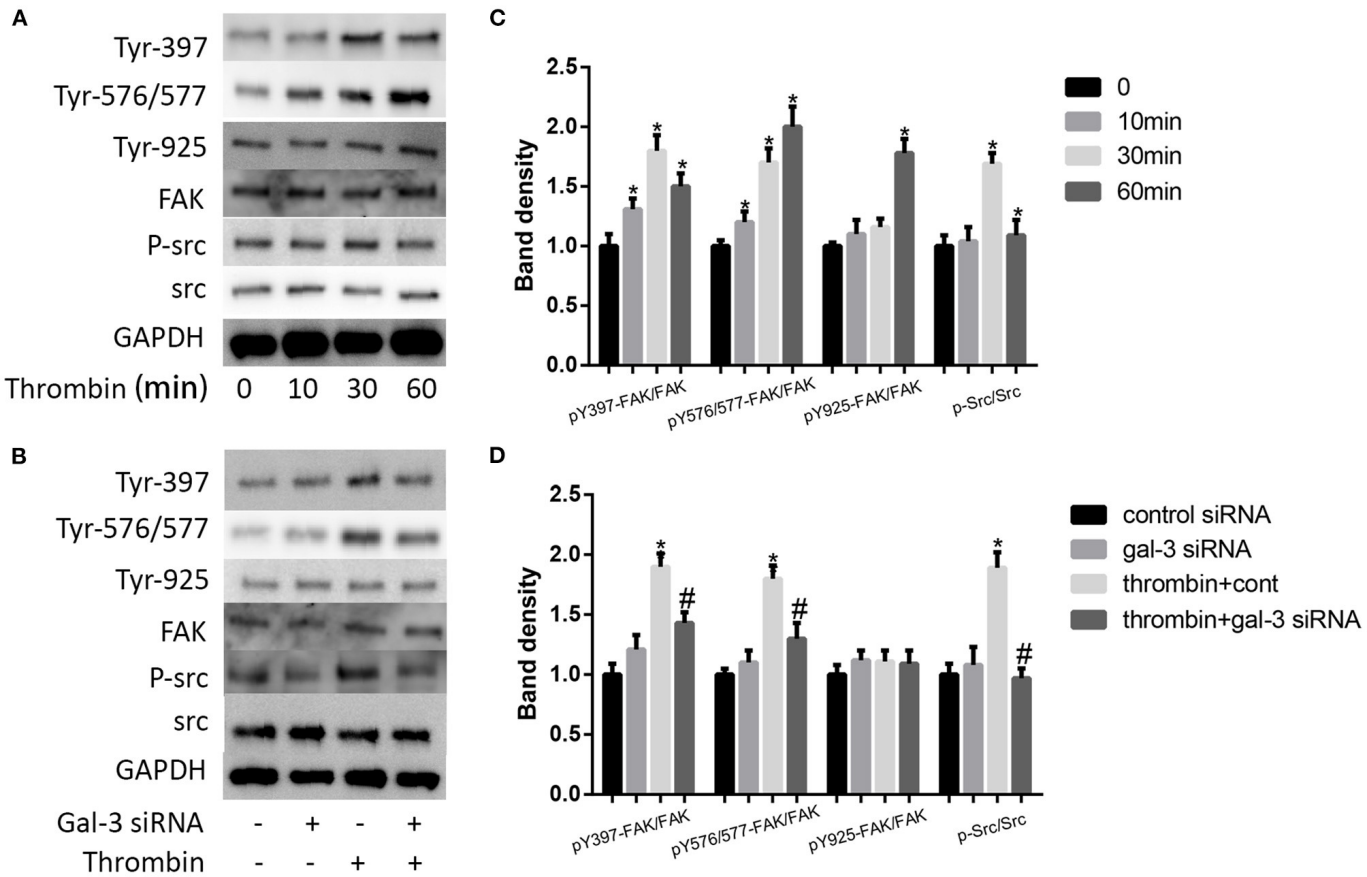
Thrombin activated distinct FAK signalings via gal-3. VSMCs were treated with thrombin over a range of concentrations (2 U/ml) for different times (0, 10, 30, and 60 min), and the expression of p-src and src, the phosphorylation of FAK at Tyr-397,−576/577, and−925, and FAK were measured by Western blot **(A,C)**. After transfection with either control or gal-3 siRNA for 24 h, VSMCs were incubated for 30 min in the absence or presence of thrombin (2 U/ml), and the expression of p-src and src, the phosphorylation of FAK at Tyr-397,−576/577, and−925, and FAK were measured by Western blot **(B,D)**. Quantification of the results is shown in the right panel. Band density of native VSMCs was defined as a control and considered to 1. *p < 0.05 vs. control; ^#^*p* < 0.05 vs. thrombin.

It has been proven that src signaling pathway and its substrate focal adhesion kinase (FAK) mediated cell migration (24). More important, FAK has been regarded as a kinase to phosphorylate paxillin. Here, we examined the impact of thrombin on FAK phosphorylation. We used 2 U/ml thrombin to deal with VSMCs for different times (0, 10, 30, and 60 min), and the expression of p-src and src, the phosphorylation of FAK at Tyr-397,−576/577, and−925, and FAK were measured by Western blot. Our results indicated that thrombin increased src phosphorylation as well as the phosphorylation of FAK at Tyr-397,−576/577, and−925; the most pronounced increase was seen on the Tyr-397 phosphorylation site (Figures 5A,C). We further analyzed the effect of gal-3 knockdown on src and FAK expression. After transfection with either control or gal-3 siRNA for 24 h, VSMCs were incubated for 30 min in the absence or presence of thrombin (2 U/ml). Compared to their control groups, gal-3-siRNA transfected cells have an obvious decreased expression tendency of phosphorylated src, Tyr-397, and Tyr-576/577; meanwhile, the phosphorylation of Tyr-925 was not significantly decreased after gal-3-siRNA treatment (Figures 5B,D). Altogether, gal-3 mediated thrombin-induced VSMC migration via the Src/FAK signaling pathway.

## Discussion

**Figure 6:**
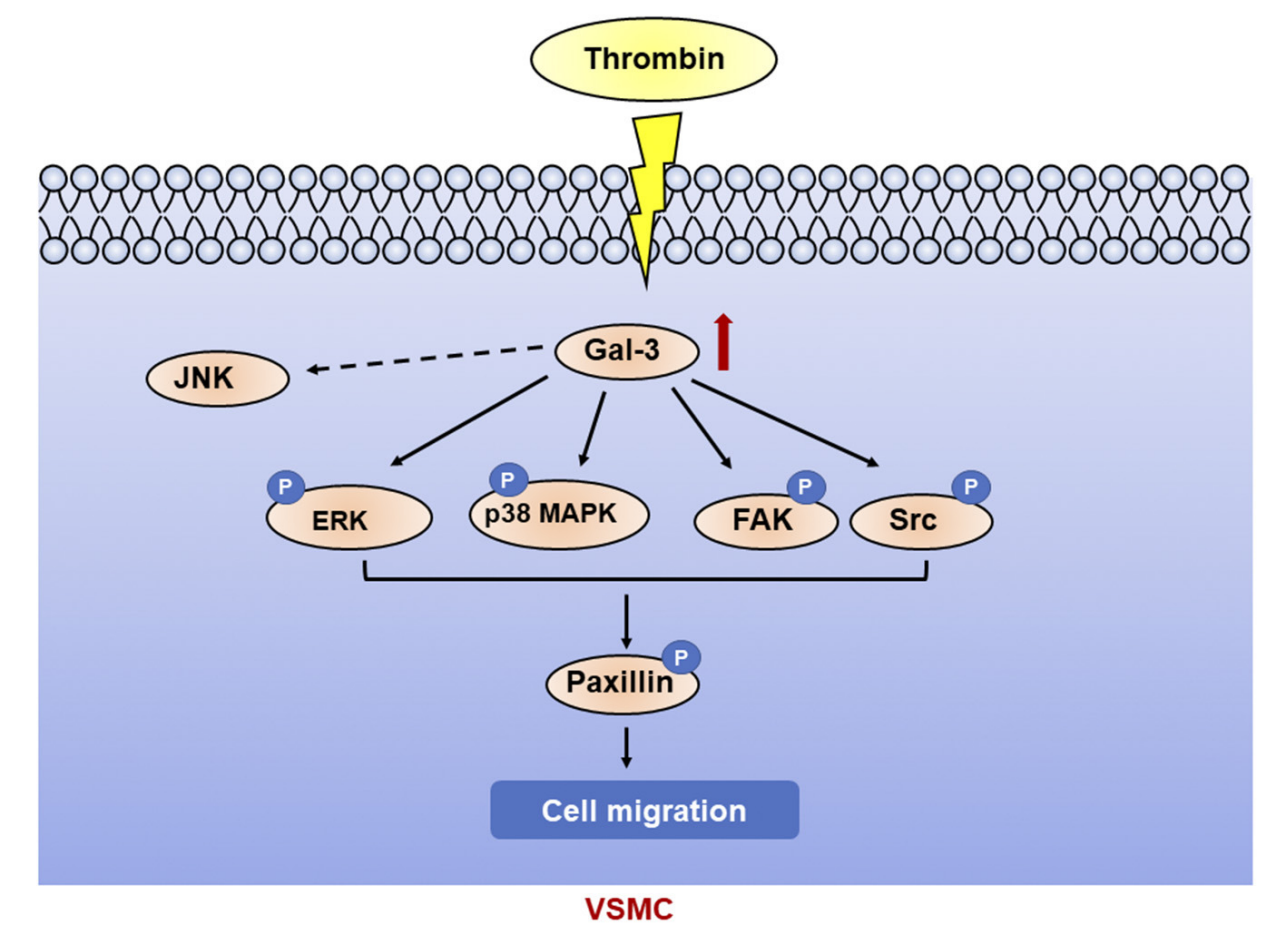
Galectin-3 mediated thrombin-induced VSMC migration. Thrombin induced activation of p38, ERK, Src, and FAK signaling pathways via gal-3, leading to paxillin phosphorylation and cell migration in VSMCs.

In this article, we found that thrombin induced migration and gal-3 expression in VSMCs. Knockdown of gal-3 by specific siRNA inhibited the activation of P38, ERK, Src, and FAK signaling pathway induced by thrombin, further prevented paxillin expression, and attenuated VSMC migration. Taken together, thrombin, a key player in the coagulation cascade, activated P38, ERK, Src, and FAK signaling pathway and mediated VSMC migration via gal-3 (Figure 6).

The VSMC migration from media to intima plays a crucial role in both physiological and pathological processes of atherosclerosis. Numerous studies have demonstrated that thrombin promotes cellular migration (25, 26). Gal-3 is an anti-adhesive factor that promotes SMC migration and may accelerate atherogenesis (16, 27). In our research, we found that thrombin increased gal-3 expression. In order to explore how these two factors get involved in cell migration, we then analyzed the role of gal-3 in thrombin-induced migration in VSMCs. VSMCs transfected with gal-3 siRNA have impaired ability of migration, even after thrombin treatment. Our results indicated that thrombin in collaboration with gal-3 promoted cell migration.

The mammalian MAPK family includes P38 MAPK,ERK and JNK, MAPK signaling pathways have been proved to play an important role in modulating cell migration (28, 29). In this study, our results indicated that phosphorylation of ERK1/2 and p38 MAPK, but not JNK, significantly activated thrombin via gal-3. Paxillin is an adaptor and scaffold protein that plays a critical role in focal adhesion and cell motility (22). In our research, gal-3 siRNA partly aborted thrombin-induced paxillin phosphorylation. Gao et al. found that ERK1/2 activation further induced the paxillin phosphorylation at tyrosine 31 (23). In VSMCs, we show that not only ERK1/2 but also P38 MAPK mediated thrombin-induced phosphorylation of paxillin. Our research revealed that P38 and ERK signaling pathway are both involved in thrombin-mediated VSMC migration.

There are three main tyrosine sites of FAK phosphorylation, including Tyr-397,−576/577, and−925, which are selectively regulated after the activation of ErbB receptors (30–33). The activation of FAK has been regarded as a key regulatory step toward cell migration. FAK, a scaffolding protein and an integral component of focal adhesions, is anchored via paxillin. FAK has also been proved to be one of the kinases to phosphorylate paxillin, which plays an important role in cell motility (23, 34). Autophosphorylation of Y397, an initial sign in the FAK activation, serves as a docking site for Src binding (35, 36). Schaller et al. also found that c-Src can interact with FAK by Tyr-397 (37). Y576/Y577 phosphorylation can be subsequently phosphorylated by Y397 results in a full FAK kinase activation (38). Here, we observed that thrombin increased phosphorylation of c-Src, Tyr-397, and Tyr-576/577; besides, gal-3 siRNA effectively decreased thrombin-induced phosphorylation of c-Src, Tyr-397, and Tyr-576/577. The phosphorylation of FAK at Y925 has been found to possibly regulate the focal adhesion disassembly and contribute to focal adhesion stability (39). However, here, phosphorylation of Tyr-925 was almost unchanged after treatment of thrombin or gal-3 siRNA.

In conclusion, our study demonstrated that thrombin induced migration via gal-3 in VSMCs, which was significantly mediated by P38, ERK, Src, and FAK signaling pathways. Gal-3 expression has been demonstrated to develop in atherosclerosis and increases with plaque severity (40). VSMCs, one of the most important cells in atherosclerosis, also express gal-3 in atherosclerotic plaque (41). VSMCs and gal-3 play an important role in a variety of pathological mechanisms of atherosclerosis. Thus, our work could help to clarify the role of gal-3 in thrombin-induced advanced lesions in atherosclerosis and shed new insights into the regulatory mechanism of VSMC migration in combating plaque rupture.

## Data Availability Statement

The original contributions presented in the study are included in the article/supplementary materials, further inquiries can be directed to the corresponding author/s.

## Author Contributions

LT, RZ, and FD substantial contributions to the conception or design of the work. LT and C-KH performed the experiments. LT, C-KH, and FD are involved in interpreting the data and provide the experimental materials. LT and RZ supervised the project and wrote the manuscript. All authors contributed to the article and approved the submitted version.

## Funding

This work was supported by Research Fund for the Cross between Medical and Engineering of Shanghai Jiaotong University (YG2021QN20).

## Conflict of Interest

The authors declare that the research was conducted in the absence of any commercial or financial relationships that could be construed as a potential conflict of interest.

## Publisher's Note

All claims expressed in this article are solely those of the authors and do not necessarily represent those of their affiliated organizations, or those of the publisher, the editors and the reviewers. Any product that may be evaluated in this article, or claim that may be made by its manufacturer, is not guaranteed or endorsed by the publisher.
